# Automated Artificial Intelligence Mapping of Coronary Plaque Calcification: A Comparison with Manual Intravascular Image Analysis

**DOI:** 10.3390/jcm14228166

**Published:** 2025-11-18

**Authors:** Killian J. McCarthy, Emily A. Larnard, Christina K. Anderson, Mohsin Chowdhury, Ronny Shalev, Diaa A. Hakim, Kevin J. Croce, Eric A. Osborn

**Affiliations:** 1 Division of Cardiovascular Medicine, Beth Israel Deaconess Medical Center, Harvard Medical School, Boston, MA 02215, USA; killian.mccarthy@advocatehealth.org (K.J.M.); ealarnar@bidmc.harvard.edu (E.A.L.); doctor.tina19@gmail.com (C.K.A.); mchowdhuryc@gmail.com (M.C.); 2 Dyad Medical Inc., Boston, MA 02134, USA; ronny@dyadmed.com; 3 Division of Cardiovascular Medicine, Brigham and Women’s Hospital, Harvard Medical School, Boston, MA 02115, USA; dhakim1@bwh.harvard.edu (D.A.H.); kcroce@bwh.harvard.edu (K.J.C.)

**Keywords:** artificial intelligence, calcium, optical coherence tomography

## Abstract

**Background/Aims:** Intravascular imaging during percutaneous coronary intervention (PCI) improves clinical outcomes; however, is dependent on accurate and rapid interpretation of the images generated. This study aimed to compare coronary artery calcification assessment using a novel automated artificial intelligence (AI) software algorithm with manual optical coherence tomography (OCT) image analysis. **Methods and Results:** A deep neural network based on a UNet-like architecture was developed and trained to identify calcified atherosclerotic plaque from an independent dataset of expert-annotated clinical intravascular OCT pullbacks. The AI network was subsequently validated on previously unseen clinical OCT pullbacks that were manually annotated for plaque calcium and used to quantify clinically relevant calcified plaque characteristics. Correlation and agreement between the expert-annotated images and the model predictions were evaluated. In total, 8259 cross-sectional images comprised the training and internal validation dataset. Pixel-based classification by the AI model performed best to identify calcified plaque (AUC = 0.96), with an overall diagnostic accuracy of 73.3%. During independent external validation, the model correctly identified 934 of the 1248 calcified plaques, corresponding to a diagnostic accuracy of 74.8%. The AI model performed well in assessing the calculated OCT-calcium score (ρ = 0.84; 95% confidence interval [CI], 0.81–0.87, *p* ≤ 0.001). **Conclusions:** Implementation of an automated AI software algorithm provides a rapid and efficient method to comprehensively map coronary calcium in intravascular OCT images. With further training and refinement, it is anticipated that the AI machine learning software will continue to improve, enabling new robust tools for clinical OCT calcium detection to better guide PCI procedures.

## 1. Introduction

Intravascular imaging overcomes the limitations of coronary angiography and improves clinical outcomes during percutaneous coronary intervention (PCI) by providing important adjunctive information for accurate vessel measurements, characterization of plaque composition, and optimization of stent implantation [1]. Despite the established clinical benefits [2,3,4,5] and support of society guidelines [6,7,8], intravascular imaging guidance during PCI remains severely underutilized in clinical practice [9]. A major barrier to more widespread clinical adoption is the specialized knowledge base required for operators to interpret intravascular imaging studies accurately and reproducibly, coupled with the large volume of images to be reviewed and synthesized into real-time decision making during the PCI procedure [10].

Optical coherence tomography (OCT) is a high-resolution intravascular imaging modality that offers detailed visualization of atherosclerotic plaques and endovascular stents [11]. In calcified plaques, OCT can measure the arc, length, and thickness of the calcified segment, key metrics with important clinical implications for lesion preparation to optimize acute stent implantation [12,13,14]. However, studies demonstrate that the clinical outcome benefits of intravascular imaging-guided PCI are primarily afforded to those who achieve optimal results with regard to lesion preparation, vessel sizing, and stent expansion/apposition (as shown in [15]), and thus data omission or misinterpretation during manual image inspection may negatively impact operator decisions, particularly in calcified lesions.

The emergence of artificial intelligence (AI) clinical decision making support algorithms is poised to enable powerful new tools for intravascular image analysis during PCI [16,17,18]. Specifically, automation of OCT calcium detection holds potential to significantly improve the efficiency and accuracy of current OCT image analysis workflows that rely on time-consuming manual image review and annotations. To address this need, we aimed to develop a data-handling and algorithm-training pipeline focused on deep learning for intravascular OCT, and then compare the results of automated AI processes to manual human OCT image analysis in the comprehensive assessment of coronary artery plaque calcification.

## 2. Methods

### 2.1. Study Design

This is a retrospective, post hoc, multicenter study designed to evaluate the accuracy and reproducibility of an AI algorithm for assessing calcified coronary plaque. De-identified intravascular OCT image data was obtained from four different US academic clinical sites with local Institutional Review Board approval by each participating institution. All OCT imaging pullbacks were performed as part of standard of care in the evaluation of obstructive coronary artery disease using clinically available OCT imaging systems. All OCT images were acquired using a commercial OCT imaging system (Abbott Vascular, Chicago, IL, USA) following the administration of intracoronary nitroglycerin at a pullback speed of 18–36 mm/s using manual contrast injection for blood clearance. Exclusion criteria included lesions with associated plaque rupture, aorto-ostial lesions, previously stented lesions, bypass graft lesions, chronic total occlusions, and pullbacks with insufficient image quality due to a lack of blood clearance or the presence of obscuring image catheter artifacts. All other datasets not meeting the exclusion criteria were included in the analysis. Two data groupings were utilized for the study: the first dataset group was employed for the development, training, and internal validation of the deep learning network architecture, and a second, different dataset was used for independent external validation via manual image analysis.

### 2.2. Ground Truth Annotation

The ground truth was generated through the manual annotation of coronary artery plaques by a human clinician with considerable experience interpreting clinical intravascular OCT pullbacks. Plaques were labeled according to composition based on consensus recommendations for OCT plaque image interpretation, including calcium, fibrous tissue, and lipids [11]. If more than one plaque type was identified on a single cross-sectional image, each plaque was labeled separately. Lumen contours and non-tissue components, including guidewire location and its shadow, were also labeled. In total, 10 different pixel classes were labeled for model development, training, and internal validation.

### 2.3. Deep Network Architecture Development and Training

**Figure 1:**
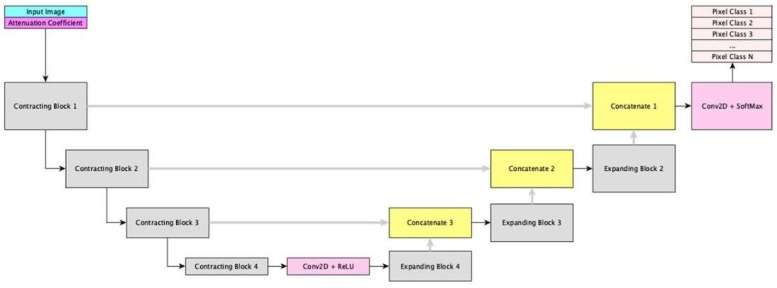
Schematic of the UNet-like architecture. The contracting (**left**) path was composed of four convolutional blocks, each comprised of two 3 × 3 2D convolutional layers and an activation function (rectified linear unit/ReLU), followed by a 2D maxpooling layer and a dropout layer connecting adjacent convolutional blocks. The expanding (**right**) path was composed of a 2D upsampling layer, which matched the corresponding 2D maxpooling layer in the contracting path, followed by a 2D convolutional layer and an activation function (ReLU). In combination with both vertical and horizontal feature bridges, a final segmentation map was produced.

A UNet-like architecture was designed, consisting of (1) a contracting path to downsample the input image and extract high-level features, (2) an expanding path to upsample the feature maps from the contracting path, and (3) vertical and horizontal feature bridges to enable concatenation to preserve spatial information and produce a final segmentation map (Figure 1). OCT image data was pre-processed prior to feeding into the contracting path to ensure all data, including manual annotation, was represented as polar coordinates (r, θ) on a log-scale. In addition, training data was augmented by using a custom augmentation function mimicking intravascular OCT artifacts such as Non-uniform Rotational Distortion (NURD), motion, multiple reflection, etc. One additional image channel was computed and appended, which is the attenuation coefficient of the original image data. The attenuation coefficient was then gamma-adjusted and scaled to fit into the 0–255 pixel intensity value range. Both channels of the image were then normalized by subtracting 127.5 and dividing by 127.5 to force the pixel values into the [−1, 1] range to avoid interference with the final network performance. The contracting path was composed of four convolutional blocks, each comprised of two 3 × 3 2D convolutional layers and an activation function (rectified linear unit/ReLU), followed by a 2D maxpooling layer and a dropout layer connecting adjacent convolutional blocks. Following experimentation with different patterns, a default of 16 filters was used for the first convolutional block, and each subsequent block had double the number of filters. In the expanding path, the convolutional block design comprised a 2D upsampling layer that matched the corresponding 2D maxpooling layer in the contracting path, followed by a 2D convolutional layer and an activation function (ReLU). Ultimately, a high-resolution final segmentation map was produced.

For deep neural network design and training purposes, the manually annotated pullbacks were randomly divided into a training dataset and an internal validation dataset to ensure all pullback data utilized for validation had not previously been seen by the AI software algorithm. Following the development of the deep neural network, a number of metrics were assessed at different epochs to optimize final network performance prior to independent external validation, including per pixel class area under the receiver operating characteristic (ROC) curve to gauge the classification accuracy of the network across a wide range of probability thresholds and plaque confusion matrix to assess the quality of pixel-level segmentation.

### 2.4. Independent External Validation

External validation of the deep neural network architecture was performed on a unique testing dataset, which was distinct from the training dataset as detailed above. All pullback data in this dataset had not been seen by the algorithm during development and internal validation. Three independent, experienced OCT readers manually annotated an equal proportion of 50 OCT pullbacks (23–24 pullbacks each), with 10 of the 50 pullbacks being annotated by all three readers to allow for assessment of intra-observer variability. Each pullback was annotated for calcified plaque at 1 mm intervals, including calcium minimum thickness (measured in mm on the cross-sectional image), calcium maximum thickness (also measured in mm on the cross-sectional image), arc (defined as the angle of calcium in degrees as referenced to a point in the center of the lumen), and length (the single longest continuous calcified segment in each individual calcified plaque measured in mm). For multiple plaques in the same frame, measurements were reported for each individual plaque. The OCT-calcium score was then calculated as the total sum of the following characteristics: maximum calcium thickness (1 point for >0.5 mm), maximum arc (2 points for >180°), and length (1 point for >5 mm) [12]. The three OCT readers performing the external testing practiced at a different center than the clinician who performed ground truth annotation for initial training and validation.

### 2.5. Statistical Analysis

Categorical variables were described using counts and proportions. Continuous variables were described using the mean and standard deviation or the median and interquartile ranges. For internal validation, sensitivity and specificity were assessed for each pixel class, and an ROC curve and area under the curve (AUC) were generated to assess the pixel class classification accuracy of the model. This allowed for a Youden Index to be computed to identify an optimal threshold. As pixel class classification accuracy is not sufficient for model selection alone, a 3 × 3 plaque confusion matrix was also generated to assess the quality of the pixel-level segmentation (precision, recall, and F1-score). For independent external validation, correlation between the three expert readers and the model predictions was evaluated using Pearson or Spearman correlation tests as appropriate. Agreement was assessed by the intraclass correlation coefficient and Dice coefficient. Importantly, 95% confidence intervals were reported where indicated. Statistical significance was denoted with a *p*-value of ≤0.001. Statistical analysis was executed using SPSS (IBM, Armonk, NY, USA).

## 3. Results

### 3.1. OCT Pullback Characteristics

A total of 197 intravascular OCT pullbacks from four different clinical sites were analyzed in the study, divided into a training and internal validation dataset and an independent, distinct external testing dataset consisting of 147 and 50 pullbacks, respectively. On average, each pullback consisted of 440 cross-sectional frames (range 374–541), which were labeled by the deep neural network. In total, 8259 cross-sectional frames were used for the training and internal validation dataset, and 5676 frames were used for the independent external testing dataset. Table 1 and Table 2 describe the baseline and procedural characteristics of the independent external testing dataset, respectively.

### 3.2. Internal Validation

The deep neural network model was assessed at a pixel level, where in total the ground truth pixel count for calcified plaque was 21,717,610, fibrous plaque was 17,342,891, and lipid plaque was 17,241,233. Performance was optimal when all three plaque morphologies had equally weighted losses. The model performed best in pixel-based classification when identifying calcified and lipidic plaque (AUC = 0.96), followed by fibrous plaque (AUC = 0.92). The optimal threshold for a Youden Index close to its maximum value was within the range of 0.08–0.1, consistent with that expected for a classifier with 10 target classes. Other pixel classes, including lumen boundary (AUC = 0.99), guidewire (AUC = 0.99), guidewire shadow (AUC = 0.95), and general tissue not representing any other pixel class (AUC = 0.95), were identified with high accuracy by the model. The model performance was also assessed through a 3 × 3 plaque confusion matrix. The sum of the diagonal components of the confusion matrix resulted in an overall accuracy of 73.3%, with the model performing best on fibrous and lipid plaque (diagnostic accuracy of 83.3%), followed by calcified plaque (diagnostic accuracy of 73.3%). There were cases of both false positives and false negatives generated by the AI software, all of which were included in the final analysis.

### 3.3. Independent External Validation

**Figure 2:**
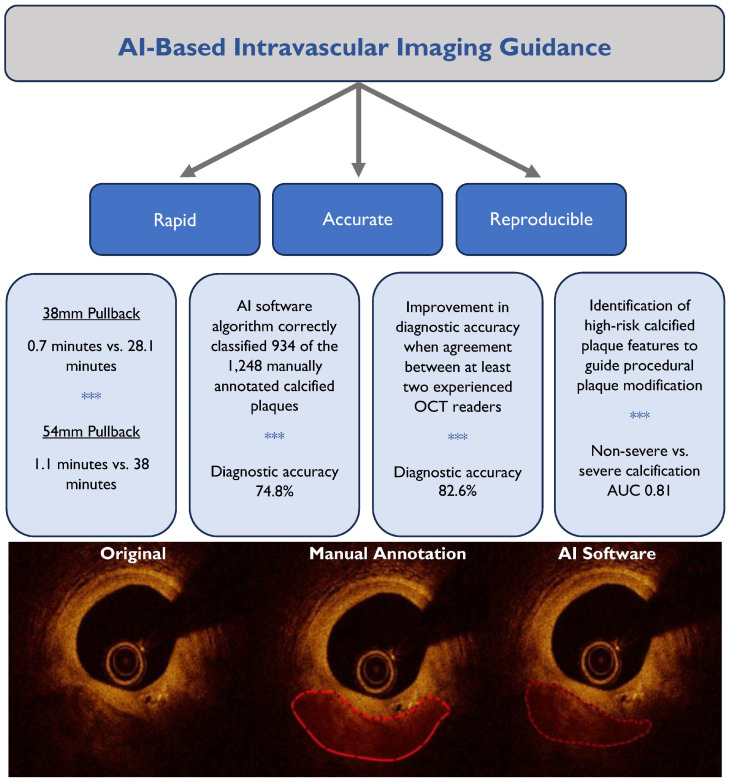
Benefits of artificial intelligence guidance for intravascular imaging optimization during PCI *** including a summary of main results of the study and examples of calcium annotation by the manual reader and the AI software algorithm.

A total of 50 OCT pullbacks (5676 cross-sectional frames) annotated at 1 mm intervals provided a total of 2422 cross-sectional frames and 1248 calcified plaques for comparison with the deep neural network model predictions (Figure 2). The median lesion calcium minimum thickness was 0.75 mm (IQR 0.58 mm, 0.97 mm), arc of calcium was 60.8° (IQR 42.4°, 87.8°), and OCT-calcium score was 1 (IQR 1, 2). The mean time required by the deep neural network model to analyze an OCT pullback of 54 mm and 75 mm in length was 0.7 min and 1.1 min, respectively, compared to 28.1 min and 38.0 min, respectively, for manual analysis. The AI software algorithm correctly classified 934 of the 1248 calcified plaques identified by manual analysis, corresponding to a diagnostic accuracy of 74.8%. 1D Dice coefficient, which assesses the angular overlap between manual analysis and the model prediction, was 0.78. In the subset of 10 OCT pullbacks annotated by all three OCT readers, when there was agreement by at least two readers, the diagnostic accuracy increased to 82.6%, with the AI software algorithm correctly characterizing 119 out of 144 calcified plaques.

**Figure 3:**
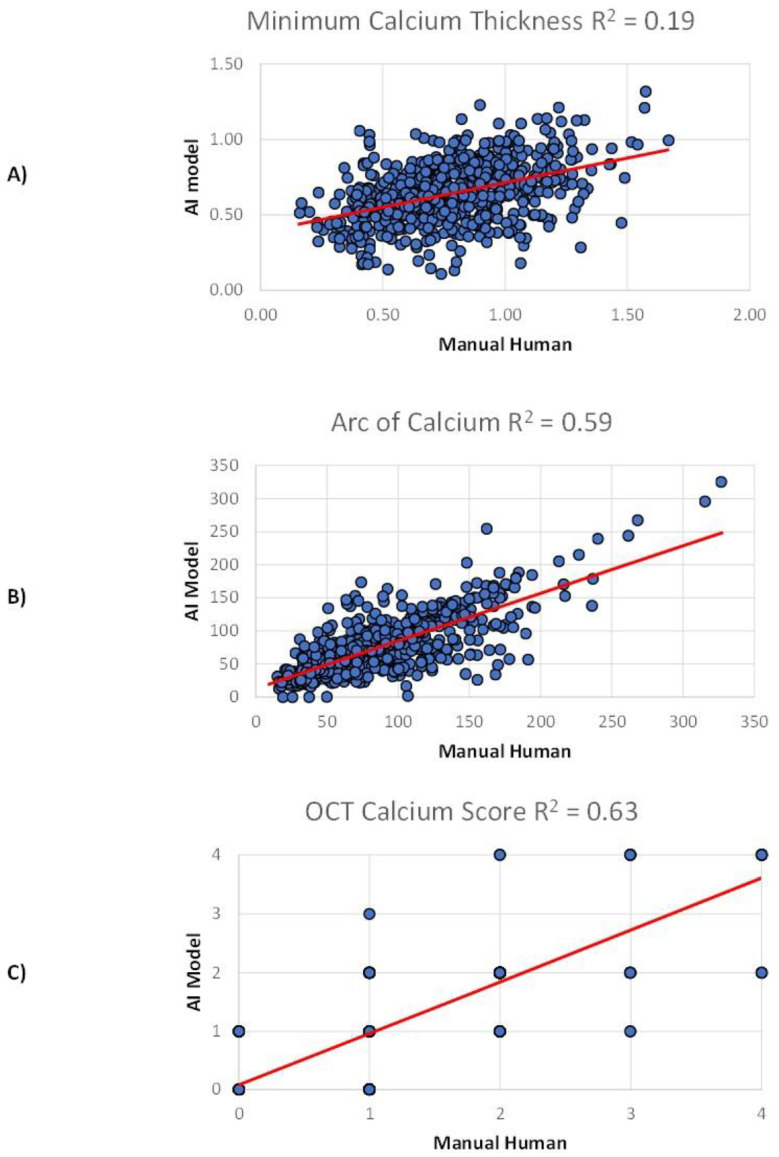
Scatterplots depicting the correlation between the experienced OCT human readers and AI model. (**A**) Minimum calcium thickness, (**B**) arc of calcium, and (**C**) OCT-calcium score. Manual human interpretation is represented on the X-axis, and the AI-model interpretation is represented on the Y-axis.

**Figure 4:**
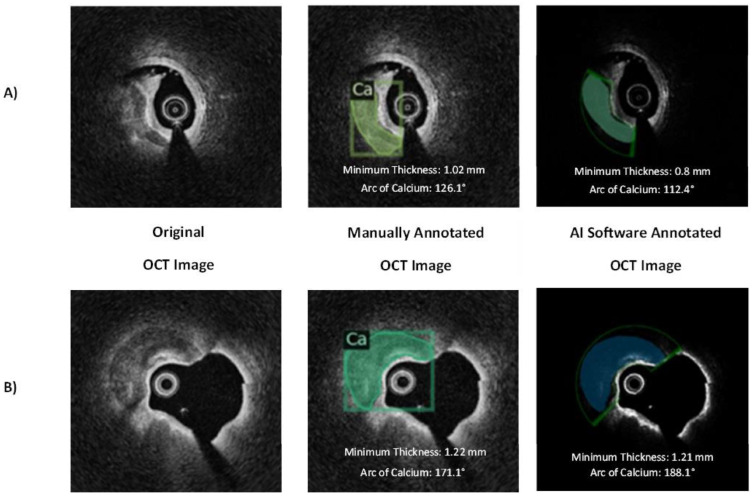
Manual segmentation vs. automated artificial intelligence analysis of calcified coronary plaques. Representative examples showing (**A**) calcium score 1 and (**B**) calcium score 3. The OCT-calcium score is calculated as the total sum of the following characteristics: calcium thickness (1 point for >0.5 mm), maximum arc (2 points for >180°), and length (1 point for >5 mm), with OCT calcium scores of >3 being associated with higher potential for clinical stent under-expansion. The first image of (**A**,**B**) depicts the unedited, original clinical OCT frame with the OCT catheter in the center of the lumen (dark black space). The second image depicts the manually annotated clinical OCT frame with calcified plaque highlighted (green) and minimum thickness and arc of calcium reported. The third image depicts the AI algorithm-annotated clinical OCT frame with calcified plaque highlighted (green/blue) and minimum thickness and arc of calcium reported. Ca = calcified plaque.

The AI model performance varied depending on the calcified plaque characteristic being analyzed. There was moderate correlation between the experienced OCT readers and the deep neural network in the assessment of the minimum thickness of calcified plaque (r = 0.44; 95% CI, 0.37–0.51, ICC = 0.53, *p* ≤ 0.001), which improved when consensus was reached between the OCT readers (r = 0.55; 95% CI, 0.38–0.68, ICC = 0.66, *p* ≤ 0.001). There was strong correlation in the assessment of the arc of calcified plaque (ρ = 0.73; 95% CI, 0.68–0.77, ICC = 0.86, *p* ≤ 0.001) and very strong correlation with OCT-calcium score (ρ = 0.84; 95% CI, 0.81–0.87, ICC = 0.88, *p* ≤ 0.001), neither of which were significantly impacted by reader consensus. Figure 3 depicts the correlation between the human OCT readers and the AI model for each calcified plaque characteristic. The OCT-calcium score was subsequently dichotomized into non-severe calcification (score 0–2) and severe calcification (3–4), with ROC curve analysis determining an AUC of 0.81. Figure 4 provides representative examples of the AI model’s and human readers’ interpretation of OCT frames showing calcified plaques of differing severity.

Assessment of intra-observer variability across the 10 OCT pullbacks annotated by all three manual OCT readers demonstrated similar findings to the correlation between manual analysis and the deep neural network model. There was a moderate correlation between the readers in the assessment of the minimum thickness of calcified plaque (r = 0.42–0.68, ICC 0.50–0.77, *p* ≤ 0.001), strong correlation in the assessment of the arc of calcified plaque (ρ = 0.61–0.79, ICC 0.75–0.80, *p* ≤ 0.001), and very strong correlation with OCT-calcium score (ρ = 0.76–0.88, ICC 0.80–0.89, *p* ≤ 0.001).

## 4. Discussion

Automated AI-driven plaque detection algorithms that comprehensively map coronary calcium hold particular clinical importance for guiding intraprocedural PCI decision making regarding calcified plaque modification with atherectomy or intravascular lithotripsy devices [19,20,21,22,23], given the strong association between optimal stent expansion and improved clinical outcomes [24,25,26]. To address this need, we aimed to develop a novel deep neural network model utilizing a UNet-like architecture that provides a rapid and efficient method to comprehensively assess calcified coronary plaque from intravascular OCT images. While several studies to date have shown promising results in examining atherosclerotic plaque and non-tissues structures with intravascular OCT [27,28,29,30,31], our study further adds to current data by demonstrating rapidly obtained results with high agreement between expert-annotated plaque calcium and model predictions (overall diagnostic accuracy of 74.8%, improving to 82.6% when there was agreement between at least two expert readers), and strong correlation with clinically relevant calcified plaque characteristics, such as the OCT-calcium score (ρ = 0.84, ICC = 0.88, AUC 0.81). Some factors that may have contributed to incorrect labeling by the AI model include OCT image quality variability leading to plaque misclassification, the presence of OCT artifacts, class imbalance in training datasets leading to underrepresentation of certain plaque subtypes, and limited training data diversity leading to incorrect labeling.

Early investigations with machine learning focused on coronary artery plaque segmentation, such as image feature extraction using Support Vector Machine, hidden Markov random field, and other legacy machine learning algorithms, suggesting improved inter-observer agreement when compared to fully manual analysis alone [30,31]. As deep learning techniques evolved, newer algorithms were successfully employed for OCT-based coronary artery analysis, including convolutional neural networks in pediatric patients with Kawasaki disease [32] and deep learning models in adults [33]. While these models performed well in the binary classification of “plaque” and “no plaque”, due to the small size of the datasets on which the AI models were trained, they were limited in their ability to distinguish between different plaque subtypes. More recently, deep learning networks involving UNet segmentation have been developed, which allow for relatively smaller training image datasets to be utilized without impacting generalizability and with reduced plaque error classification [34]. Chu et al. performed the largest study to date utilizing a U-shaped encoder–decoder architecture in intravascular OCT image analysis for automatic plaque characterization [35]. Internal validation showed excellent detection of atherosclerotic plaques of all types, with a diagnostic accuracy of 97.6% for fibrous plaque, 90.5% for lipidic plaque, and 88.5% for calcified plaque. External validation showed a similarly high overall diagnostic accuracy of 86.6% when consensus was reached between the three core laboratories. Overall, the results provided further evidence of the role artificial intelligence can play to reduce subjectivity in OCT image interpretation, and in 2021, the first clinically available AI software for intravascular OCT calcium detection, Ultreon, was released by Abbott Vascular with promising early data [36,37,38]. Since then, Gentuity HF-OCT and Hypervue Spectrawave have developed and made commercially available their own OCT AI software algorithms. While there are many similarities between these and our software, we believe the addition of calcified plaque characteristic analysis (length, minimum and maximum thickness, and arc of calcium, allowing an OCT calcium score to be generated) provides additional information beyond simple plaque identification to help guide and optimize PCI.

In several ways, the AI model used in our study has similarities to that developed by Chu et al. as both models (1) resemble a UNet architecture, consisting of a contracting and expanding pathway and vertical and horizontal feature bridges to allow for better preservation of spatial information, (2) apply cross-entropy loss and the Dice coefficient to allow for better segmentation of pixels and more accuracy in automatic plaque characterization and differentiation, and (3) were trained and validated on clinical intravascular OCT pullbacks, encompassing a diversity of atherosclerotic plaque types and lesion complexity. However, we further enhanced the current algorithm’s ability to generalize results in two integral ways. First, we improved data augmentation to resemble relevant clinical artifacts by creating non-uniform rotational distortion (NURD) augmentation. Second, we broadened the U-Net architecture’s input to include hand-crafted features extracted (e.g., attenuation coefficient) in addition to traditional expert segmentation. With these key algorithm enhancements, our model demonstrates that the generalization of analyses improves significantly.

While both models were trained to characterize atherosclerotic plaque composition, our study provides further clinically relevant advances by assessing specific calcified plaque characteristics, such as calcium thickness, arc of plaque calcium, length of plaque calcium, and OCT-calcium score. While maximum calcium thickness forms part of the OCT-calcium score, we chose to also report minimum calcium thickness as an independent measurement, given its clinical relevance regarding the potential for balloon-mediated calcium fracture. Unfortunately, there was only moderate correlation between the AI software and manual reader with regard to minimum calcium thickness, likely due to pixel-level calcified plaque detection with the AI software identifying smaller segments of calcified plaque not visible to the human reader. As decision making regarding the need for plaque modification at the time of PCI is of critical importance to mitigate the risk for stent under-expansion and poor clinical outcomes in severely calcified lesions [24,25,26], the capability of the AI algorithm tested in this study to automatically map coronary calcification offers a new, powerful clinical tool for intravascular OCT imaging PCI guidance.

## 5. Limitations

As the intravascular OCT pullback dataset utilized for AI model training and validation excluded stented vessels and unstable lesions exhibiting plaque rupture, our results are not generalizable to these populations. In addition, the ground truth annotation in our study was performed by a single experienced OCT reader, limiting intra-observer variability during training and internal validation of the AI software algorithm. The measurement of calcified plaque length differed for the AI software and manual reader; while the AI software interrogated every frame of each OCT pullback for adjacent calcified plaque, the manual reader examined every 1 mm (5 frames) for contiguous calcium in the same quadrant and therefore may have underestimated very short calcified segments. Although we reported calcium modification techniques used and minimum stent areas post-PCI, unfortunately, there was too few data to evaluate correlations and outcomes related to calcified plaque characteristics for these variables. This will be an important area for future research. Finally, due to the intrinsic properties of near-infrared light penetration into complex atherosclerotic tissue, the posterior aspect of the calcified segment was not always able to be identified, impacting the assessment of calcified plaque thickness.

## 6. Conclusions

Integration of a deep learning neural network for intravascular OCT image analysis provides a rapid and efficient method to automatically map calcified coronary artery atherosclerotic plaque during PCI procedures and calculate clinically relevant characteristics, such as the OCT calcium score, which can be used to guide optimal vessel preparation and stent optimization. With further training and refinement, it is anticipated that the AI software algorithm will continue to improve, enabling new robust tools for clinical intravascular OCT calcium detection.

## Figures and Tables

**Table 1:** Baseline characteristics of the independent external testing dataset.

Characteristic	Total (N = 50)
Sex—No. (%)	
Male	37 (74)
Age—years	
Median	64.5
Range	[35, 89]
Race—No. (%)	
Caucasian	38 (76)
African American	6 (12)
Other	6 (12)
Smoking—No. (%)	
Current	9 (18)
Former	19 (38)
Comorbidities—No. (%)	
Hypertension	34 (68)
Dyslipidemia	37 (74)
Diabetes Mellitus	13 (26)
Atrial Fibrillation	3 (6)
Peripheral Arterial Disease	1 (2)
Coronary artery disease	19 (38)
Prior MI	10 (20)
Prior PCI	11 (22)
Prior CABG	5 (1)
Renal function—median (range)	
Creatinine (g/dL)	1.0 (0.6, 10.9)
eGFR (mL/min/1.73 m^2^)	74.25 (4.3, 136.1)
Dialysis (No., %)	1 (2)
LVEF—%	
Median	57.1
Range	30, 75
Medications—No. (%)	
Aspirin	28 (56)
P2Y12 inhibitor	7 (14)
DOAC	2 (4)
Warfarin	1 (2)
Statin	29 (58)
ACE inhibitor/ARB/ARNI	15 (30)
Beta blocker	24 (48)
Calcium channel blocker	13 (26)
Insulin	3 (6)
Oral diabetic agent	8 (16)
Clinical presentation—No. (%)	
Silent ischemia	2 (4)
Stable angina	19 (38)
Unstable angina	11 (22)
NSTEMI	12 (24)
STEMI	6 (12)

MI—myocardial infarction, PCI—percutaneous coronary intervention, CABG—coronary artery bypass graft, DOAC—direct oral anticoagulant, ACE—angiotensin-converting enzyme, ARB—angiotensin II receptor blocker, ARNI—angiotensin receptor-neprilysin inhibitor, NSTEMI—non-ST elevation myocardial infarction, STEMI—ST elevation myocardial infarction.

**Table 2:** Procedural characteristics of the independent external testing dataset.

Characteristic	Total (N = 50)
Duration—median (range)	
Procedure (mins)	91 (33, 191)
Fluoroscopy (mins)	18.2 (9, 60.8)
Radiation dose—mGy	
Median	667
Range	[239, 2772]
Contrast volume—mL	
Median	147.5
Range	(80, 360)
Access—No. (%)	
Radial	44 (88)
Ulnar	1 (2)
Femoral	5 (10)
PCI—No. (%)	46 (92)
OCT—No. (%)	
Pre-PCI	50 (100)
Post-PCI	40 (80)
Vessel OCT performed—No. (%)	
LAD	37 (74)
LCx	4 (8)
RCA	8 (16)
Other	1 (2)
Minimum lumen area—mm^2^	
Median	1.79
Range	[0.5, 4.8]
Calcified plaque characteristics	
Minimum thickness, mm—median [range]	0.72 [0.16, 1.66]
Arc of calcium, °—median [range]	66.95 [9.30, 327.20]
OCT-calcium score—median [range]	1 [0, 4]
Calcium modification performed—No. (%)	
Rotational atherectomy	5 (10)
Laser atherectomy	0 (0)
Intravascular lithotripsy	2 (4)
Cutting/Scoring balloon angioplasty	2 (4)
Minimum stent area post PCI—mm^2^	
Median	5.12
Range	[2.91, 9.95]

PCI—percutaneous coronary intervention, OCT—optical coherence tomography, LAD—left anterior descending artery, LCx—left circumflex artery, RCA—right coronary artery.

## Data Availability

The original contributions presented in this study are included in the article. Further inquiries can be directed to the corresponding author.

## Abbreviations

PCI	Percutaneous Coronary Intervention.
OCT	Optical Coherence Tomography.
AI	Artificial Intelligence.
AUC	Area under the Receiving Operator Characteristic Curve.
NURD	Non-uniform Rotational Distortion.
ROC	Receiver Operating Characteristic Curve.
